# A major *Calanus finmarchicus* overwintering population inside a deep fjord in northern Norway: implications for cod larvae recruitment success

**DOI:** 10.1093/plankt/fbw024

**Published:** 2016-05-30

**Authors:** Boris Espinasse, Sünnje L. Basedow, Vigdis Tverberg, Tore Hattermann, Ketil Eiane

**Affiliations:** 1 Faculty of Biosciences and Aquaculture, University of Nordland, Bodø N-8049, Norway; 2 Akvaplan-Niva As, Strandgata 11, Tromsø 9007, Norway

**Keywords:** fjord-shelf exchange, sub-polar ecosystem, particle tracking, Lofoten coast

## Abstract

High *Calanus finmarchicus* abundances were recorded in wintertime in Vestfjorden, close to the main cod breeding grounds off Lofoten and Vesterålen, northern Norway. The mean abundance for locations with water depth >500 m was ∼37000 ind. m^−2^ (range: 26700–49000 ind. m^−2^). To our knowledge, this is the first report of massive overwintering of *C. finmarchicus* on the Norwegian shelf. Because of the observed size and location of this population, we argue that local overwintering on the northern Norwegian shelf can contribute significantly to sustain a *C. finmarchicus* population on the shelf during the period of first feeding for cod larvae. This is supported by a particle tracking model.

## INTRODUCTION

The North Atlantic and subarctic holoplanktonic calanoid copepod *Calanus finmarchicus* is a key food source for many marine animals including the larval stages of cod (*Gadus morhua*) (Planque and Batten, 2000; Falk-Petersen *et al*., 2007, 2009; Heath and Lough, 2007). In the main cod spawning areas on the Lofoten and Vesterålen shelves, off the coast of northern Norway, complex hydrography accumulates both early cod larvae and their main prey, the naupliar stages of *C. finmarchicus* (Ellertsen *et al*., 1984). This spatiotemporal match between nauplii and cod larvae abundances is important for recruitment to the Arcto-Norwegian cod stock (Klungsøyr *et al*., 1989). Inter-annual variations of the main fish stock depend, in part, on larval recruitment success, which is closely linked to prey availability (Sundby, 2000; Head *et al*., 2005).

The spring distribution of *C. finmarchicus* nauplii reflects spawning events occurring immediately after the seasonal ascent migration from the deep hibernation habitats, where the copepodite stage V (CV) dwells for overwintering (Broms *et al*., 2009; Bagøien *et al*., 2012; Head *et al*., 2013). The deep water hibernation strategy reduces loss rates due to predation and advective dispersal during winter, but when the population ascends into surface waters and copepodites molt to the sexually mature adult stages in late winter or spring (Melle *et al*., 2014), they are subject to high predation risk and surface circulation that may disperse its members over large distances (Halvorsen *et al*., 2003; Gaardsted *et al*., 2010). Thus, the early spring shelf population of *C. finmarchicus* nauplii is assumed to be advected in from oceanic populations, where highest abundances have been recorded (Slagstad and Tande, 1996; Halvorsen *et al*., 2003).

Deep fjords may also provide suitable overwintering habitats for *C. finmarchicus* on the Norwegian shelves, but the relatively low abundances observed there in winter (Skreslet and Rød, 1986; Bagøien *et al*., 2001; Skreslet *et al*., 2015) suggest that many such populations may not be a significant food source for populations of cod larvae relative to the oceanic overwintering populations.

We investigated whether overwintering populations of *C. finmarchicus* in a deep fjord basin close to historically important cod breeding grounds are large enough to significantly contribute to the origin of the shelf population; and if the main circulation pattern can lead to advection of this population toward the coast of the Lofoten.

## METHOD

**Fig. 1.**
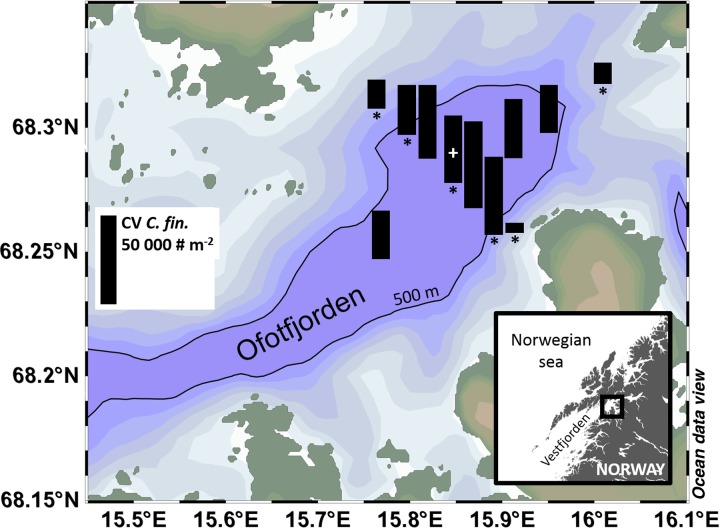
Map of the study area with the location of the sampling stations. The height of the black bar represents the abundance of copepodite the Stage V of *Calanus finmarchicus* based on the reference bar shown in the left part of the figure. Manual counting was done of samples from stations marked with a star and additional net tows were conducted at the central station (white +) for different depth layers. The 500-m depth isobath is indicated.

The study site was Ofotfjorden, the inner part of Vestfjorden (surface area ∼400 km^2^, maximum bottom depth ∼615 m; Fig. 1). Ofotfjorden does not have a proper sill, but the separation between Vestfjorden and the inner Ofotfjorden is bottleneck shaped, with the deep part of the bathymetry (deeper than 100 m) narrowing from 20 to 2 km in width, and widening again to 8 km. The circulation in the upper layers of Vestfjorden is characterized by the presence of eddies influenced both by wind conditions and bathymetric steering (Mitchelson-Jacob and Sundby, 2001). Vestfjorden is also a main cod spawning ground and a critical habitat for cod larvae survival (Ottersen *et al*., 2014).

Zooplankton samples were collected by vertical net hauls from bottom to surface using a WP2 net (180-µm mesh size, mouth area=0.25 m^2^) at 11 stations in the inner part of Vestfjorden (Fig. 1 for station locations), during 20–21 January 2015 aboard R/V “Tanteyen.” The WP2 was equipped with a CTD-F (SD204, SAIV A/S) mounted on the frame. Additional net tows in the surface layer (upper 100 m) were done at the central station. Net samples were fixed in a ∼4% formaldehyde in seawater solution. *Calanus finmarchicus* copepodites were staged and counted in the laboratory for selected stations (6 samples+4 at the central station, see Fig. 1). Abundance and size structure (not shown in this paper) of zooplankton communities were estimated using an adapted scanner coupled with an image analysis software, the ZooScan/ZooProcess system (described in Gorsky *et al*., 2010). CV abundances were estimated from the size class from 1.5 to 2 mm equivalent spherical diameter and validated by manual counts (Manual_count= 0.87 × ZooScan_count, *R* ^2^= 0.96, *n* = 10).

## RESULTS AND DISCUSSION

CV represented over 90% of the *C. finmarchicus* at all the stations, while CIVs, the second most abundant stage, accounted for 2–7%. The highest estimated abundances of development stage CV were encountered in the deepest part of the fjord and peaked at ∼49 000 ind. m^−2^ (Fig. 1). The mean abundance at stations with water deeper than 500 m was 37 000 ind. m^−2^ [standard deviation (SD) = 7780, *n* = 7], compared with 15 100 ind. m^−2^ (SD = 8200, *n* = 4) for shallower stations. Very low abundances (∼290 ind. m^−2^) were measured in the upper 100 m at the central station.

A rough estimation of the *C. finmarchicus* stock in our study area (Fig. 1) was done computing the areas deeper and shallower than 500 m, respectively, and corresponding mean abundances detailed in the text above. This resulted in a stock size of 2.45 × 10^12^ individuals which is equivalent to a carbon biomass of 333 t (assuming 0.136 mg C ind.^−1^; Campbell *et al*., 2001). This is high compared with the estimated stock size of cod larvae from Bogstad *et al*. (Bogstad *et al*., 2015) as assuming that 40% of the cod eggs were spawned in the Lofoten area (Ellertsen *et al*., 1987), the cod larvae biomass estimated in this area is ∼9.4 t C (assuming a C weight of 0.056 mg ind.^−1^ (Ellertsen *et al*., 1987) and using C: dry weight ratio of 0.4).

The limited data available in the literature suggests that the mean abundance of overwintering *Calanus* in Norwegian coastal waters tends to be between 10 000 and 12 000 ind. m^−2^ (Table I). In comparison, data from the shelf break and oceanic locations off the Lofoten shelf are generally higher with mean values about 25–30 000 ind. m^−2^ and maximum values up to 150 000 ind. m^−2^. Our data indicate abundances in the study area were comparable with those previously found off-shelf and higher than those found in the fjords (mean of 29 000 and 37 000 ind. m^−2^ for the deepest stations).

**Table I:** Historical data collection of CV *Calanus finmarchicus* abundances in the Norwegian Sea and the adjacent fjords during winter

Study site	Date	No. of Stn. (samples)	Mean ind. m^−2^ (min–max)	Publication
Fjords–North Norwegian coast	1977	2 (4)	10 100 (900–28 200)	Skreslet and Rød, 1986
	1997–1998	1 (3)	12 600 (10 500–14 100)	Skreslet *et al*., 2000
	1983–2005	2 (23)	10 300 (390–38 400)	Skreslet *et al*., 2015
	2015	11 (11)	29 000 (5200–48 900)	This study
Fjords—South Norwegian coast	1995–1996	4 (9)	12 530 (2920–33 200)	Bagøien *et al*., 2001
Off-shelf—Lofoten basin	2000–2002	58 (128)	27 400 (<2000–150 000)	Halvorsen *et al*., 2003
	2003–2004	∼50 (141)	26 300	Edvardsen *et al*., 2006

The origin of this population occurring in the inner part of Vestfjorden is not yet completely understood. A part of the population is likely advected from the South by the main circulation, but local production may also contribute. Recent studies provided evidence of a second spawning period around August in coastal waters of the Norwegian Sea (Bagøien *et al*., 2012). It seems plausible that *C. finmarchicus* from this second peak, experiencing a different current circulation than in spring, can be trapped in the inner part of the fjord where they would remain for overwintering. Indeed, changing hydroclimatic conditions over the seasons result in different circulation patterns on the shelf. During winter, the combination of weak run-off and south-easterly prevailing winds tend to retain copepods in deep fjords (Skreslet and Rød, 1986), thus contributing to the aggregation of *C. finmarchicus*. Furthermore, the location of the *C. finmarchicus* population in the deeper layer of the water column, where weak currents occur, limits loss of individuals by advection. This is confirmed by our observational data showing high abundances of overwintering *C. finmarchicus* in the deeper area of the inner part of Vestfjorden.

**Fig. 2.**
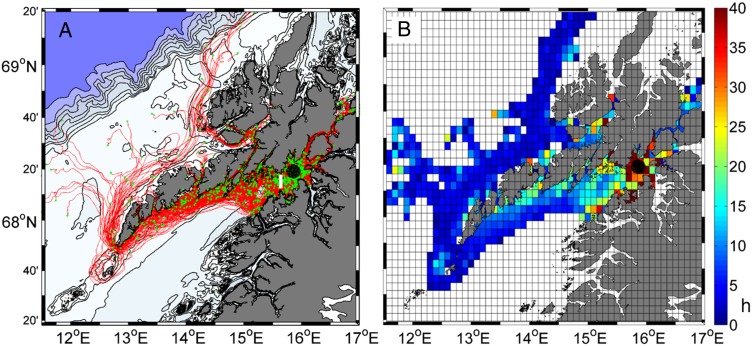
Particle trajectories (**A**) and the mean time of residence of the particles in hours (**B**) obtained by simulations with a particle tracking model (IHCTHYOP) and based on modeled current velocity fields from ROMS3D/NorKyst800. One thousand particles (black dots) were released on 15 March 2011 in the upper 50 m of a 10-km-diameter disk, and simulated for a duration of 20 days. In (A), green dots show the position of the particles at the end of the 20-day simulation period, and the thick white line indicate the border between the inner and the outer part of Vestfjorden. In (B), the color coding indicates the average time spent, in hours, by particles in each grid cell during the simulations.

To assess if this fjord population can potentially be advected out of the inner part of Vestfjorden to the Lofoten coast, we used a particle tracking approach. Velocity fields were provided from a run of the ocean modeling system NorKyst-800, which has been developed using ROMS-3D structure. A complete description of NorKyst-800 including all relevant references is provided by Albersten *et al*. (Albersten *et al*., 2011). The Norkyst-800 model has been used with success in other recent studies showing relatively good agreement with field data (e.g. Myksvoll *et al*., 2014; Skarðhamar *et al*., 2015). In summary, bathymetric data (50 m resolution) comes from the Norwegian Mapping Authority Hydrographic Service; the Norwegian Meteorological Institute's (MET) Nordic-4 km provided lateral boundary conditions; the atmospheric forcing is based on MET's weather forecast model HIRLAM-12 km; tidal forces were based on a global inverse barotropic model of ocean tides (TPXO7.2) and Norwegian river discharges were modeled by the Norwegian Water Resources and Energy Directorate. The model domain covered the geographical area from 65° to 71.7° N and 5.5° to 21.3° E, resulting in a grid of 420 × 800 cells of 800 m separation. A spin-up period exceeding 1 year was used and the output fields of the model were saved every hour. The particle trajectories were computed with a free Lagrangian transport modeling system, ICHTHYOP (www.ichthyop.org), designed to study how physical and biological factors affect plankton dynamics. A full description of the model can be found in Lett *et al*. (Lett *et al*., 2008). A patch of 1000 particles, simulating nauplii, was released uniformly in a 10-km diameter disk (Fig. 2). The particles were homogenously distributed in the upper 50 m of the water column where nauplii are mainly distributed (Skreslet *et al*., 2000). Each particle was passively advected, without any depth constraints, over a 20-day simulation period starting on 15 March 2011, thus providing the position of nauplii at the beginning of April, which roughly corresponds with the period of high concentrations of cod larvae in this area. The year 2011 has been chosen here because simulated velocity field data for 2015 are not available yet. This simulation is shown as an example and does not intend to be representative of average conditions.

The trajectories show that the main path used by the particles lead them from the inner part of Vestfjorden to the southeastern coast of the Lofoten islands (Fig. 2A). Very few particles (1.6%) were advected into the narrow coastal fjords and 4.6% of them drifted northward into the straights east to Vesterålen Islands. After 20 days, 55.2% of the particles were advected out of the inner part of the fjord and some particles had started to move northward along the northwestern coast of the Lofoten after passing around the tip of the archipelago. At the end of the simulation, 83.7% of the particles were still distributed in the upper 60 m. The particles were advected relatively slowly from the inner part of Vestfjorden (5–8 cm s^−1^; Fig. 2B). Once outside of Ofotfjorden, the particles closest to the Lofoten coast (northern side of the fjord) were advected at moderate speed (8–13 cm s^−1^) whereas particles in the central part of the fjord or the ones which reached the northwest coast moved very quickly (22–97 cm s^−1^).

The flushing of the fjord water surface layer in spring is promoted by prevailing northerly winds (offshore oriented Ekman transport) and increased fresh water run-off (Skreslet and Rød, 1986; Skarðhamar *et al*., 2007). From early spring, *C. finmarchicus* reach the surface layer for spawning and feeding on the phytoplankton bloom (Melle *et al*., 2014). At this time, the nauplii stages that generally live in the surface layer can potentially be advected out of the inner fjord region. Simulated trajectories for 2011 show that this advective process is effective in the surface layer, and that a large part of the nauplii population produced in the inner part of Vestfjorden during early spring will likely end up on the shelf around the Lofoten islands.

This part of the Norwegian shelf is the major spawning ground of Arcto-Norwegian cod, and the interaction between the circulation system and copepod phenology may explain why this region has successful cod spawning (Sundby and Nakken, 2008). The earlier development of the nauplii cohort originating from overwintering *C. finmarchicus* inside Vestfjorden compared with cohorts originating from oceanic areas further off-shelf (Pedersen *et al*., 2000) means that cod larvae developing on the shelf around the Lofoten grow in an environment with high abundances of their principal food for an extended period of time. Recent research on the match–mismatch hypothesis indicates that the duration of overlap between favorable prey concentrations and the predators might be more relevant for recruitment success and year-class strength than the overlap of peak abundances of prey and predator (Durant *et al*., 2005; Kristiansen *et al*., 2011).

## CONCLUSIONS

Both the size and the location of the overwintering *C. finmarchicus* stock in the present study are interesting in relation to a potential role in sustaining a shelf population, especially along the southeastern coast, upstream of the main cod spawning grounds off Lofoten and Vesterålen. Our findings indicate that the combined input of *C. finmarchicus* nauplii and adults ready to spawn on the shelf could be substantial and significant for the success of cod larvae in April. However, further field investigations are required to describe the origin of this fjord population and to establish to what extent this population is crucial for cod larvae recruitment success.

## FUNDING

This work was funded by the ConocoPhillips Calanus project (NSBU-107021). Funding to pay the Open Access publication charges for this article was partly provided by Nord University.
